# From Prenatal Exposure to Adult Sexual Dysfunction: The Impact of Plastic-Derived Endocrine Disruptors on Testosterone Homeostasis and Erectile Function

**DOI:** 10.3390/toxics14080673

**Published:** 2026-07-29

**Authors:** Sofoklis Stavros, Panagiotis Christopoulos, Eriketi Kokkosi, Efthalia Moustakli, Athanasios Zikopoulos, Anastasios Potiris, Maria Anastasia Daskalaki, Ioannis Arkoulis, Ismini Anagnostaki, Ioanna Vassilaki, Nikolaos Kathopoulis, George Daskalakis, Peter Drakakis

**Affiliations:** 1 Third Department of Obstetrics and Gynecology, University General Hospital “ATTIKON”, Medical School, National and Kapodistrian University of Athens, 12462 Athens, Greece; apotiris@med.uoa.gr (A.P.); jeannettevas@windowslive.com (I.V.); pdrakakis@hotmail.com (P.D.); 2 Second Department of Obstetrics and Gynecology, “Aretaieion” Hospital, Medical School, National and Kapodistrian University of Athens, 11528 Athens, Greece; panchrist@med.uoa.gr; 3 Department of Midwifery, Faculty of Health and Caring Sciences, University of West Attica, 12243 Athens, Greece; ekokkosi@uniwa.gr; 4 Department of Nursing, School of Health Sciences, University of Ioannina, 45500 Ioannina, Greece; 5 Department of Reproductive Medicine and Surgery, University College London Hospitals NHS Foundation Trust, London NW1 2BU, UK; thanzik92@gmail.com; 6 First Department of Obstetrics and Gynecology, Alexandra Hospital, Medical School, National and Kapodistrian University of Athens, 11528 Athens, Greece; anastasia.daskalaki00@gmail.com (M.A.D.); garkoylis@hotmail.com (I.A.); nickatho@med.uoa.gr (N.K.); gdaskalakis@yahoo.com (G.D.); 7 Medical School, National and Kapodistrian University of Athens, 11528 Athens, Greece; isanagnostaki3@gmail.com

**Keywords:** male reproductive health, steroidogenesis, HPG axis, Leydig cells, oxidative stress, developmental origins of health and disease, microplastics, nanoplastics, environmental exposure, endocrine disruption

## Abstract

Bisphenols, phthalates, and other plastic-associated compounds are endocrine-disrupting chemicals (EDCs), common environmental contaminants that can disrupt hormonal homeostasis. Human exposure to such chemicals occurs mainly through food packaging, consumer goods, medical devices, and the environment, starting right from early development stages up to adulthood. These chemicals might cause damage to male reproductive systems as a result of being anti-androgens and estrogenic chemicals. However, the proper development of the male reproductive system requires well-regulated hormonal signaling pathways; hence, exposure to such compounds during the developmental stages poses a great risk. Environmental exposure to plastics during fetal development may influence the development of the testes, reduce the function of the Leydig cells, disrupt steroidogenesis, and produce epigenetic modifications, as documented through studies. This has been shown to increase the risk of developing reproductive disorders and reduce the production of testosterone. Therefore, one of the pathophysiological links between endocrine disruptor exposure and ED might be testosterone deficiency. Apart from disrupting testosterone production, the plastic-sourced EDCs could influence various physiological processes associated with erectile performance, such as those related to vasculature, inflammation, metabolism, and endocrinology. In this review, a comprehensive overview of the scientific data on the effect of plastic-based EDCs on testosterone regulation and male reproductive health has been provided. The role of developmental programming, endocrine disruption, oxidative stress, epigenetics, and vascular dysfunction has been explored in detail. Moreover, the possible involvement of micro- and nanoplastics has also been reviewed. From the data that is currently available, there seems to be a physiologically plausible association between plastic-based contaminants, testosterone dysregulation, and adverse reproductive outcomes.

## 1. Introduction

The production of plastics has risen dramatically over the past few decades, revolutionizing consumer products, industrial applications, medical technologies, and food preservation [1]. Over hundreds of millions of tons of plastic are already produced annually worldwide, and this amount is expected to rise over the next several decades. Despite the substantial social and economic benefits associated with the widespread use of plastics, growing concerns have emerged regarding the potential adverse effects of plastic-related environmental pollution on human health [2].

The plastic-associated substances of serious concern include endocrine-disrupting chemicals (EDCs). These chemicals represent a diverse group of compounds that can interfere with normal endocrine signaling [3]. The commonly known EDCs found in plastics include bisphenol A (BPA), which are closely related to bisphenol S (BPS) and bisphenol F (BPF) [4]. Phthalates also constitute a broad spectrum of plastic-associated chemicals used to impart flexibility to plastic products. Exposure to plastic-associated EDCs can occur through ingestion, inhalation, dermal absorption, and clinical practices [5,6]. Human studies have reported the presence of several of these chemicals in various biomatrices, including urine, blood, amniotic fluid, placenta, milk, and semen [7,8,9]. Although this review focuses on plastic-derived endocrine-disrupting chemicals, it should be recognized that endocrine disruption also results from exposure to a broader range of environmental pollutants, including airborne contaminants. These pollutants may enter the body through inhalation or dermal absorption and have been associated with adverse reproductive, developmental, and metabolic outcomes through mechanisms involving hormonal dysregulation. Consequently, plastic-derived EDCs should be considered within the broader context of cumulative environmental exposures (exposome) affecting endocrine and reproductive health [10].

The male reproductive system seems to be especially susceptible to endocrine disruption. Normal male sexual development, fetal testes development, puberty, spermatogenesis, and adult male sexual function require proper androgen signaling [11,12]. The hypothalamus–pituitary–gonad (HPG) axis may change as a result of certain EDCs from plastic acting as anti-androgens, estrogens, or inhibitors of steroidogenesis, according to experimental research. This may contribute to Leydig cell dysfunction, altered testosterone synthesis, disrupted androgen receptor signaling, oxidative stress (OS), and epigenetic changes. Most significantly, exposure during delicate developmental phases may have long-term biological effects [13,14,15].

The idea of developmental programming, also known as the Developmental Origins of Health and Disease (DOHaD) hypothesis, is therefore quite popular. This theory suggests that exposure to certain environmental factors during development may cause persistent changes in organs and their physiological functions [16,17]. Many studies in animal models proved that prenatal exposure to bisphenols and phthalates causes underdevelopment of testicles, low levels of testosterone, and negative effects on the reproductive system in adult life of the offspring. Similar results were obtained when human subjects were studied, though not as consistently as in animals [18,19].

Testosterone is vital for optimal sexual function and erectile physiology in addition to its critical role in fertility. Libido, nitric oxide (NO) generation, endothelial function, smooth muscle tone, and penile vascular responsiveness all depend on normal androgen levels. As a result, erectile dysfunction (ED), a disorder that impairs quality of life and may be an early indicator of underlying vascular disease, has been linked to testosterone deprivation [20,21]. Environmental contaminants may affect erectile function through multiple mechanisms, including OS, inflammation, metabolic dysfunction, vascular dysfunction, and disrupted androgen signaling [22,23].

Concerns about the potential effects of plastic exposure on human reproductive health have increased in recent years, particularly because of the growing recognition of microplastics and nanoplastics as emerging environmental contaminants. These concerns are related to the biological properties of these particles, including their ability to induce oxidative stress, inflammation, tissue damage, and cellular dysfunction, as well as their capacity to act as carriers of EDCs [15,24,25,26].

While several evaluations have looked at male infertility, reproductive toxicity, and endocrine disruptors independently, very few have combined these linked topics into a unified conceptual framework. Furthermore, there are still few thorough reviews that address these subjects in a cohesive way, despite the research on microplastics, endocrine disruption, and male reproductive and sexual health growing quickly. This narrative review focuses on the impact of plastic-derived endocrine disruptors on testosterone homeostasis and erectile function, with particular emphasis on the mechanistic pathways linking endocrine disruption, developmental programming, and male reproductive health. Prenatal programming, OS, epigenetic regulation, and micro- and nanoplastics are discussed within this conceptual framework as biological mechanisms that may contribute to testosterone dysregulation and erectile dysfunction.

## 2. Literature Search Strategy

This paper offers a literature review that comprehensively examines the current scientific evidence concerning the impacts of plastic-related endocrine disrupters on testosterone levels, male fertility, and erectile dysfunction. A comprehensive literature search was performed using PubMed/MEDLINE, Scopus, and Web of Science to identify relevant studies published through June 2026.

The search strategy combined Medical Subject Headings (MeSH) and free-text terms related to endocrine disruption, plastics, testosterone, and erectile dysfunction. Keywords included combinations of: “endocrine-disrupting chemicals”, “EDCs”, “bisphenol A”, “BPA”, “bisphenol S”, “BPS”, “bisphenol F”, “BPF”, “phthalates”, “plasticizers”, “microplastics”, “nanoplastics”, “testosterone”, “androgens”, “Leydig cells”, “steroidogenesis”, “male reproductive health”, “male infertility”, “sexual dysfunction”, and “erectile dysfunction”. Boolean operators (AND, OR) were used to optimize search sensitivity and specificity.

Experimental studies in animal models, mechanistic studies investigating hormonal pathways, epidemiological studies, and review articles published in English were examined for this review. Studies investigating plastic-derived endocrine-disrupting chemicals and their effects on testosterone homeostasis, developmental programming, male reproductive health, and erectile dysfunction were considered eligible for inclusion. Conference abstracts, editorials, letters to the editor, non-English publications, duplicate records, and studies not directly relevant to the scope of this review were excluded. Additional relevant publications were identified through a manual search of the references of the selected articles. Particular emphasis was placed on studies addressing prenatal and postnatal exposure, developmental programming of reproductive physiology, hormonal regulation of testosterone production, and mechanisms linking endocrine disruption to ED.

When multiple publications addressed similar topics, priority was given to recent high-quality evidence, including systematic reviews, meta-analyses, well-designed epidemiological studies, and mechanistic investigations that provided substantial biological insight. Where overlapping evidence was identified, duplicate findings were not reported separately, and preference was given to the most comprehensive or up-to-date publications. Studies reporting conflicting or negative findings were also considered and are discussed where relevant to provide a balanced overview of the available evidence.

Given the interdisciplinary nature and broad scope of the topic, the purpose of this review was to provide a comprehensive narrative synthesis of the available knowledge and not to conduct a systematic review or meta-analysis. Therefore, no formal assessment of the risk of bias of individual studies nor quantitative synthesis of the data was performed.

Figure Preparation: The graphical abstract and conceptual figures were developed by the authors to summarize the biological mechanisms discussed in this review. ChatGPT (OpenAI, GPT-5.5) was used to assist in generating preliminary conceptual layouts during figure development. The final figures were subsequently redesigned, substantially revised, and manually edited by the authors using standard graphical software. All scientific content, figure organization, labeling, and graphical elements were critically reviewed and verified by the authors, who assume full responsibility for the accuracy and originality of the final figures.

## 3. Plastic-Derived Endocrine Disruptors: Sources and Human Exposure

### 3.1. Bisphenols

The bisphenols form part of the extensively studied endocrine disruptors derived from plastics. Bisphenol A is a chemical compound utilized in the production of polycarbonate plastics and epoxy resins that are usually found in products such as food and drink containers, water bottles, thermal paper, medical equipment, and lining for metal cans [4,27]. Due to growing concerns regarding the endocrine-disrupting properties of BPA, its use has been progressively replaced by structurally similar analogues, including BPS and BPF. However, recently there have been studies showing that these substitutes may possess endocrine-disrupting properties as potent as BPA’s or even more potent [28,29].

The mode of action for bisphenols includes interacting with androgen and estrogen receptors as well as other nuclear receptors to elicit biological effects. The bisphenols BPS and BPF have demonstrated endocrine-disrupting activity, posing safety concerns over the substitute materials [30,31].

### 3.2. Phthalates and Plasticizers

Phthalates represent yet another important group of EDCs, which can be frequently detected in food packages, toys, floorings, cosmetics, and medical equipment, such as pipes, as well as in some medications. They are commonly used as plasticizers, which increase the toughness and flexibility of plastic compounds. The absence of chemical bonds between phthalates and the polymer matrix allows them to be released from plastic products [32,33,34].

The most well-known types of phthalates are butyl benzyl phthalate (BBP), dibutyl phthalate (DBP), and di-(2-ethylhexyl) phthalate (DEHP). Several scientific studies have associated phthalate exposure with undesirable reproductive effects. In particular, anogenital distance shortening, dysfunction of the testicles, malformations of the developing male reproductive system, and reproductive disorders in the future have been found to be associated with phthalate exposure during fetal development [35,36].

### 3.3. Microplastics and Nanoplastics

Microplastics, nanoplastics, and plastic additives have recently become prominent contributors to environmental pollution. Microplastics are usually defined as small plastic particles less than 5 mm in size, while nanoplastics can be defined as very small plastic particles that might have unique biological properties because of their size and high specific surface area. The particles can be manufactured or produced from the breakdown of bigger plastic products [37,38].

The particles have been found in food, drinking water, indoor air, human blood, placenta, human breast milk, marine environment, and reproductive tissues. Besides being carriers of heavy metals, persistent organic pollutants, endocrine disruptors, and other types of environmental pollutants, the particles may have some adverse impacts on biological systems directly [39,40,41]. There is an increasing body of research evidence indicating that micro- and nano-plastic particles may negatively influence endocrine and reproductive functions. Moreover, they may amplify the biological activity of endocrine active substances [42,43].

### 3.4. Routes of Human Exposure and Bioaccumulation

Human beings can come into contact with EDCs from plastics via ingestion, inhalation, dermal contact, and medical devices or procedures [44]. Ingestion is one of the most significant pathways of exposure and happens through ingestion of food or drinks packed or preserved in plastic products. Human skin can be exposed to EDCs from plastics through cosmetics and thermal paper, while inhalation of plastics is yet another major pathway of exposure [45].

Nevertheless, exposure can happen even prior to conception. The blood of mothers, placenta, amniotic fluid, and cord blood were found to be contaminated with bisphenols, phthalates, and microplastics. It shows that it is possible for these substances to permeate biological barriers and affect the developing fetus [46,47,48]. The development of fetuses is a very delicate process characterized by carefully regulated hormonal signals and rapid proliferation of cells; therefore, it is particularly vulnerable to the influence of any environmental exposures. In this regard, exposure during this critical stage of development may lead to reproductive problems in the future [49,50].

Many studies have found that there is plastic contamination in people around the world. Even though many of these contaminants are eliminated from the body fairly quickly, since they persist in the environment and people are continually exposed to them, this has caused researchers to be concerned about the effects that they could have on fertility and hormone levels. It is vital that we understand the ways in which these contaminants affect males [11,51,52,53].

## 4. Mechanisms of Endocrine Disruption in the Male Reproductive System

EDCs derived from plastics act as disruptors of the endocrine system through multiple biological mechanisms that have been investigated primarily in in vitro experiments and experimental animal models. Human evidence supporting these mechanisms is derived mainly from observational epidemiological studies, as direct investigation of molecular events within reproductive tissues is not feasible. Consequently, the strength of evidence differs across the proposed mechanisms discussed below [54,55,56,57].

### 4.1. Disruption of the HPG Axis

The HPG axis is the primary endocrine regulatory system governing male reproductive development and testosterone production. The hypothalamus secretes gonadotropin-releasing hormone (GnRH), which causes the anterior pituitary to secrete luteinizing hormone (LH) and follicle-stimulating hormone (FSH). While FSH regulates Sertoli cell activity and supports spermatogenesis, LH acts on Leydig cells in the testes to stimulate testosterone production [58,59].

Multiple EDCs have been implicated in the dysregulation of the HPG axis. Bisphenols and phthalates may affect hypothalamic secretion, alter gonadotropin activity, and interfere with endocrine feedback mechanisms that regulate hormonal homeostasis [13,14,60]. In vitro studies using hypothalamic and pituitary cell models, together with experimental animal studies, have demonstrated that BPA and certain phthalate derivatives can disrupt hypothalamic and pituitary endocrine signaling. Experimental animal studies have further shown alterations in circulating concentrations of GnRH, LH, FSH, and testosterone, indicating disruption of endocrine feedback mechanisms. Although these findings have been consistently observed in experimental models, evidence in humans remains less conclusive. Human epidemiological studies have reported associations between EDC exposure and alterations in reproductive hormone concentrations; however, the magnitude and direction of these associations vary across populations, likely reflecting differences in exposure assessment, study design, and residual confounding. This can lead to impaired testicular development and functioning and hormonal imbalance in the future [61,62].

Disruption of HPG-axis signaling during early development may result in long-term alterations in endocrine regulation. If interference occurs at this point, it is likely to result in long-term alterations in the way the HPG axis functions [13,63].

### 4.2. Impaired Steroidogenesis and Leydig Cell Dysfunction

Suppression of testosterone production is among the most consistently reported consequences of exposure to plastic-derived EDCs. Testosterone biosynthesis occurs in Leydig cells through the transport and conversion of cholesterol into androgenic hormones via the steroidogenic pathway. Several EDCs have been noted to affect this process [64,65,66].

In vitro studies using Leydig cell models and experimental animal studies suggest that BPA, phthalates, and related compounds reduce the expression and activity of key steroidogenic proteins, including StAR, CYP11A1, 3β-HSD, and 17β-HSD [18,67,68].

Beyond their effects on steroidogenesis, EDCs may induce structural and functional damage in Leydig cells through OS, impaired mitochondrial function, and inflammatory mediators. Consequently, structural and functional damage to Leydig cells may impair testosterone synthesis and contribute to androgen deficiency, findings that have been consistently reported in experimental animal models exposed to bisphenols and phthalates [11,26,54,69]. While impairment of steroidogenic pathways is among the best-characterized mechanisms identified in experimental studies, direct confirmation in humans remains limited. Most human epidemiological evidence relies on circulating hormone measurements rather than direct assessment of testicular steroidogenic activity, making causal interpretation challenging.

### 4.3. Androgen Receptor Interference

Androgen signaling depends not only on adequate testosterone levels but also on the proper functioning of androgen receptors (ARs) following the binding of testosterone or dihydrotestosterone (DHT). Following ligand binding, ARs regulate the transcription of numerous genes involved in male sexual differentiation, reproductive tract development, spermatogenesis, and sexual function [70,71].

The anti-androgenic activity of several EDCs made from plastic materials is linked to the disruption of AR signaling. Some EDCs can compete with endogenous hormones for receptor binding, whereas others may alter AR expression, transcriptional activity, or downstream signaling pathways. It should be noted that due to their anti-androgenic characteristics and capacity to suppress androgen-dependent gene expression, phthalates have been the subject of much investigation [30,72,73].

Exposure to EDCs during pregnancy may interfere with AR-mediated signaling, which might lead to issues with masculinization and reproductive tract development. In adults, disturbances in AR signaling may lead to the development of fertility disorders, sexual dysfunctions, and erectile dysfunction despite normal testosterone levels [54,72]. Current evidence for androgen receptor interference is supported predominantly by in vitro mechanistic studies and experimental animal models. Human epidemiological studies provide only indirect support through associations with altered reproductive hormone profiles and reproductive outcomes, while direct clinical evidence remains lacking.

### 4.4. OS and Mitochondrial Dysfunction

The role of OS in EDCs-induced reproductive toxicity has been extensively studied. The physiological production of ROS is necessary for proper functioning at a cellular and reproductive level. In contrast, excessive ROS production can overwhelm the body’s antioxidant defenses, leading to oxidative damage of biomolecules, including lipids, proteins, and nucleic acids [74,75,76].

The administration of EDCs, including BPA, phthalates, microplastics, and nanoplastics, has been shown in several studies to enhance OS in reproductive organs. Excessive ROS formation has been associated with mitochondrial dysfunction, apoptosis, and cellular injury in testicular tissues. As mitochondrial integrity is essential for cellular energy production and steroid hormone biosynthesis, oxidative damage may further compromise testicular function [26,77].

Given their essential role in energy metabolism and cellular homeostasis, mitochondria are particularly susceptible to the effects of EDCs. By activating inflammatory pathways, OS can exacerbate tissue damage within the reproductive tract [78,79,80]. OS is one of the most consistently reported mechanisms underlying EDC toxicity and is supported by extensive in vitro and experimental animal evidence. Nevertheless, OS represents a common downstream response to numerous environmental exposures and disease processes. Consequently, its specific contribution to plastic-derived endocrine disruption in humans remains difficult to isolate.

### 4.5. Epigenetic Modifications

EDCs from plastics may affect epigenetic modifications without altering the DNA code, apart from their endocrine action. The field of epigenetics involves several processes, such as DNA methylation, histone modifications, chromatin remodeling, and non-coding RNA-based gene regulation [81,82,83].

There have been several reports on the occurrence of epigenetic changes within the reproductive organs as a result of exposure to EDCs during the earlier stages of life. This might lead to changes in gene expression associated with steroid synthesis, hormones, reproduction, and metabolism. It is important to note that some of these changes might persist in adulthood [84,85].

The potential effects of exposure to EDCs on future generations is yet another area of investigation that is important [86]. According to experimental studies, epigenetic modifications caused by EDCs can be inherited by the next generation; thus, it may lead to the development of fertility problems in individuals who did not come into contact with the particular environmental factor. Experimental evidence from cell culture systems and animal models provides increasing evidence that plastic-derived EDCs may induce epigenetic modifications capable of persisting across generations. However, convincing evidence for transgenerational inheritance in humans remains limited, and the extent to which these experimental observations translate to human reproductive health is currently uncertain [87,88,89,90].

Collectively, the available evidence indicates that plastic-derived EDCs may interfere with male reproductive endocrinology through multiple interconnected mechanisms, including disruption of the HPG axis, impaired steroidogenesis, androgen receptor interference, OS, mitochondrial dysfunction, and epigenetic regulation [91,92]. However, the strength of evidence differs substantially across these mechanisms. While many pathways have been consistently demonstrated in in vitro experiments and experimental animal models, confirmation in humans relies predominantly on observational epidemiological studies. Direct clinical investigations of these molecular mechanisms remain largely unavailable because of the inherent limitations of studying endocrine disruption in human reproductive tissues. Consequently, these mechanisms should be regarded as biologically plausible pathways linking plastic-derived EDC exposure to reproductive dysfunction rather than definitively established causal mechanisms in humans.

A summary of the principal mechanisms through which plastic-derived EDCs disrupt male reproductive function, along with their major molecular targets and biological consequences, is presented in Table 1.

## 5. Prenatal Exposure and Developmental Programming of Male Reproductive Health

There is mounting evidence that one of the most vulnerable times for environmental endocrine disruptors to cause disruption is during pregnancy. During fetal development, organogenesis and the formation of the male reproductive system are regulated by precisely coordinated hormonal signaling pathways [78,93,94]. Whether exposed to EDCs during such sensitive developmental stages, permanent disruption of these processes may result, making an individual more susceptible to reproductive diseases later in life. The idea of EDCs influencing health is supported by the DOHaD hypothesis [95,96].

Plastic-derived chemicals, including bisphenols, phthalates, and microplastic-associated contaminants, have attracted considerable attention because of their ability to cross physiological barriers and disrupt endocrine function during fetal development. Experimental evidence derived from in vitro placental models and animal studies provides substantial evidence that prenatal exposure to plastic-derived EDCs can disrupt reproductive development. Human epidemiological studies have also reported associations between prenatal exposure biomarkers and adverse reproductive outcomes; however, findings remain heterogeneous, and the causal contribution of individual chemicals remains difficult to establish because of differences in exposure assessment, timing of sampling, and potential confounding factors [93,97].

### 5.1. Placental Transfer of Plastic-Derived Endocrine Disruptors

To regulate nutrition delivery, hormonal signaling, and fetal protection, the placenta serves as the primary point of contact between the mother’s and the fetus’s circulations. However, several environmental chemicals can cross the placenta and enter the growing fetus [98,99].

Human biomonitoring studies have confirmed that plastic-derived contaminants can cross the placental barrier and reach the developing fetus. It is important to note that environmental pollutants can cross the placental barrier, even at relatively low exposure levels [100].

Experimental studies, including in vitro placental models and animal studies, suggest that EDCs may also impair placental function by altering hormone production, nutrient transport, oxidative stress regulation, and inflammatory pathways [50,101,102]. Together, all these alterations may affect the hormonal milieu in utero. Although placental transfer of several plastic-derived contaminants has been demonstrated in biomonitoring studies, the extent to which measured fetal exposure translates into clinically meaningful reproductive outcomes remains uncertain. Furthermore, most human studies quantify exposure biomarkers rather than direct biological effects, limiting mechanistic interpretation.

### 5.2. Fetal Testicular Development and Masculinization

In the process of embryo formation, there are elaborate hormonal mechanisms that regulate the development of the male reproductive system [58]. During sex differentiation, the development of the testis begins the secretion of INSL3 and testosterone. These hormones are vital in the development of the male reproductive system and the masculinization process. The masculinization programming period is an important period in this process [58,103,104].

Disruption in the functioning of the androgens during this stage can have a significant effect on the development of the reproductive organs. Experimental animal studies have demonstrated that prenatal exposure to phthalates and BPA reduces testosterone production, impairs Leydig cell differentiation, and alters the expression of genes involved in steroidogenesis [67,105].

Numerous developmental abnormalities, including decreased anogenital distance, cryptorchidism, hypospadias, and abnormal reproductive tract development, have been observed in animal models following prenatal exposure to EDCs [106,107,108]. These developmental abnormalities have been consistently observed in experimental animal models and support the biological plausibility that prenatal EDC exposure may interfere with normal male reproductive programming. However, comparable evidence in humans remains more limited because direct assessment of fetal testicular development is not feasible, and epidemiological studies rely on indirect developmental markers such as anogenital distance and reproductive hormone profiles.

### 5.3. Developmental Origins of Testosterone Deficiency

A particularly important effect of prenatal EDC exposure might be the programming of testosterone production. Testosterone is produced by Leydig cells, which begin to develop during pregnancy and continue to support androgen levels far into adulthood [107,109,110].

Exposure to EDCs during critical developmental periods may alter the number, structure, and function of Leydig cells. Experimental animal studies have shown that prenatal EDC exposure results in reduced Leydig cell steroidogenic capacity, decreased expression of genes involved in testosterone biosynthesis, and lower androgen concentrations [11,111,112].

There are several potential explanations for the EDC’s programming of androgen production. These include altered steroidogenic enzyme gene expression, mitochondrial malfunction, elevated OS, epigenetic abnormalities, and disturbances to the regulation of the HPG system [54,113,114].

Findings from human epidemiological studies have been more heterogeneous than those from animal studies. Nevertheless, several epidemiological investigations have reported associations between EDC exposure and changes in reproductive hormone levels, reduced anogenital distance, delayed reproductive maturation, and impaired testicular function in males [91,115,116,117]. However, these associations have not been consistently observed across all populations, reflecting differences in study design, exposure assessment, timing of exposure measurement, and potential residual confounding. Consequently, although the available evidence supports biological plausibility, the long-term impact of prenatal EDC exposure on testosterone regulation in humans remains incompletely understood.

### 5.4. Long-Term Reproductive Consequences in Offspring

The effects on development from exposure to EDCs before birth could last long after pregnancy and even into adulthood. There is growing evidence that endocrine disturbance during fetal development can have long-term effects on reproductive system health. Experimental evidence suggests that prenatal exposure to plastic-derived EDCs may increase susceptibility to reproductive dysfunction later in life. Human epidemiological studies have reported associations with impaired semen quality, altered reproductive hormone profiles, and subfertility; however, these findings remain inconsistent and do not establish causality [107,118].

This information has led to the creation of the Testicular Dysgenesis Syndrome (TDS) theory, which is based on the notion that abnormalities in the development of the testicles during fetal development could lead to many types of reproductive disease, such as cryptorchidism, hypospadias, poor semen quality, infertility, and even testicular cancer. While many factors contribute to TDS, one possible factor is exposure to environmental EDCs [118,119].

Moreover, apart from fertility consequences, EDCs may impact other features of male sexual and reproductive well-being. Persistent low testosterone levels could have a substantial effect on the onset of puberty, sexual function, metabolism, and cardiovascular functioning. Since testosterone is essential for the maintenance of erectile capacity, any disturbances of androgen synthesis observed at the developmental stage may increase susceptibility to androgen deficiency-related sexual dysfunction later in life [120,121].

Evidence for transgenerational epigenetic inheritance is derived predominantly from experimental animal models. Whether similar mechanisms contribute meaningfully to human reproductive disease remains uncertain and requires confirmation in longitudinal multigenerational studies [93]. Experimental animal studies have demonstrated that offspring of EDC-exposed animals may exhibit reproductive abnormalities despite not being directly exposed, suggesting the possibility of epigenetic transgenerational inheritance. Although evidence for comparable transgenerational effects in humans remains limited, this represents an important area for future investigation [89,122,123].

Overall, evidence from experimental models supports the concept that prenatal exposure to plastic-derived EDCs may influence developmental programming of male reproductive health. Human epidemiological studies provide supportive but less consistent evidence, and direct clinical investigations remain unavailable because these developmental processes cannot be assessed experimentally in humans [50,62,121].

**Figure 1:**
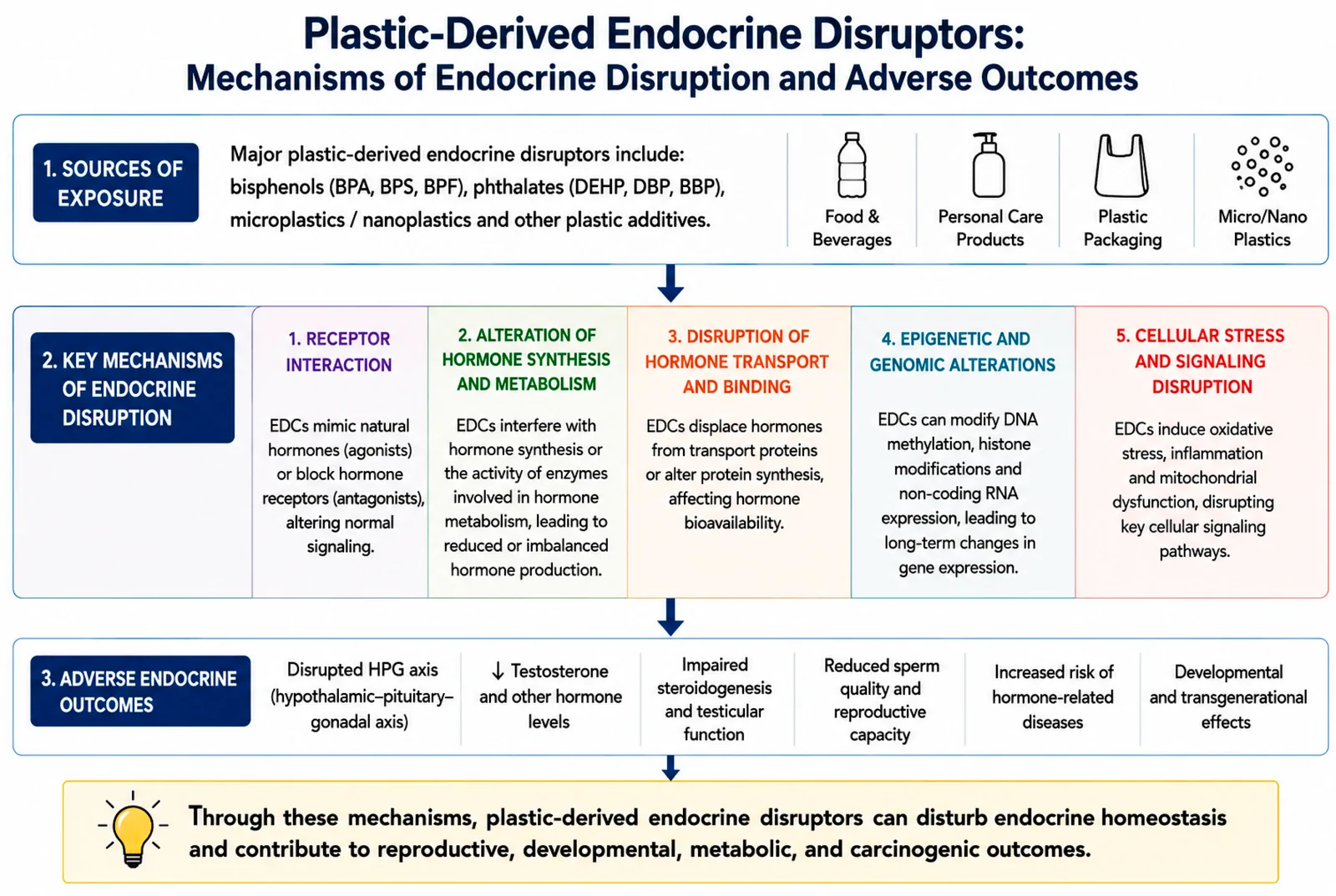
Major mechanisms through which plastic-derived endocrine-disrupting chemicals interfere with endocrine function. Plastic-derived EDCs may disrupt hormonal homeostasis by mimicking or antagonizing endogenous hormones, impairing hormone synthesis and metabolism, altering hormone transport and bioavailability, inducing epigenetic modifications, and promoting oxidative stress and cellular dysfunction. These mechanisms may contribute to HPG-axis dysregulation, impaired steroidogenesis, altered reproductive hormone levels, reduced reproductive function, and long-term reproductive and developmental consequences.

The principal developmental stages affected by prenatal exposure to plastic-derived EDCs and their potential long-term reproductive consequences are summarized in Table 2 and Figure 1.

## 6. Plastic-Derived Endocrine Disruptors and Testosterone Dysregulation Across the Lifespan

Testosterone is the primary male sex hormone and plays a critical role in reproductive development, sexual function, body composition, metabolism, and overall health [124]. Because testosterone regulation is highly dependent on endocrine homeostasis, many scientists have tried to establish a link between EDCs derived from plastics and testosterone levels. Accumulating evidence suggests that plastic-derived EDCs may interfere with testosterone regulation in both experimental models and human populations. However, the strength of evidence differs substantially between research domains. While experimental studies consistently demonstrate adverse effects on testosterone biosynthesis and endocrine signaling, findings from human epidemiological studies are more heterogeneous and are subject to important methodological limitations, including exposure misclassification, mixed chemical exposures, and residual confounding [15,125].

### 6.1. Evidence from In Vitro and Experimental Animal Studies

Some of the strongest evidence linking plastic-derived EDC exposure with alterations in testosterone secretion originates from in vitro studies and experimental animal models. Compared with human epidemiological studies, these experimental approaches provide greater control over exposure timing, dosage, and investigation of the underlying biological mechanisms [107,126].

In vitro studies using Leydig cell cultures and experimental animal studies have shown that BPA exposure is associated with decreased testosterone production. Similar findings have also been reported for BPA substitutes, including BPS and BPF. Experimental animal studies have further demonstrated alterations in the expression of steroidogenic genes, including StAR, CYP11A1, CYP17A1, and 17β-HSD, which are essential for testosterone biosynthesis [127,128,129,130].

Experimental animal studies consistently demonstrate that phthalates exert anti-androgenic effects through disruption of steroidogenesis and androgen signaling [131,132].

The growing body of research on microplastics and nanoplastics has heightened concerns about their potential impact on reproductive health. In vitro studies and experimental animal models have demonstrated that microplastics and nanoplastics can accumulate within reproductive tissues and impair reproductive function [133,134]. Although the topic has not yet been extensively investigated, available evidence suggests that microplastics may decrease testosterone secretion and contribute to reproductive dysfunction [135,136]. Collectively, findings from in vitro experiments and experimental animal studies provide strong biological plausibility that plastic-derived contaminants may impair testosterone homeostasis. However, extrapolation of these findings to humans should be undertaken cautiously because experimental exposure conditions often differ from real-world environmental exposure.

### 6.2. Evidence from Human Epidemiological Studies

Human epidemiological studies comprise several complementary lines of evidence, including biomonitoring studies documenting population exposure to plastic-derived EDCs, observational studies examining associations with circulating reproductive hormone concentrations, and prospective cohort studies evaluating developmental endocrine outcomes. Collectively, these investigations have examined whether environmental exposure to plastic-derived EDCs is associated with altered testosterone concentrations and reproductive hormone profiles. Although findings have been heterogeneous and causality cannot be inferred from most observational studies, several epidemiological investigations have reported associations between higher exposure to plastic-derived endocrine-disrupting chemicals and alterations in testosterone concentrations and other reproductive hormone profiles [7,107,137].

Several biomonitoring studies have demonstrated widespread exposure to bisphenols and phthalates in populations worldwide [138,139]. Several cross-sectional and prospective studies have reported inverse associations between urinary or serum BPA concentrations and circulating testosterone levels. Nevertheless, results have not been entirely consistent across studies. Some investigations have reported weak or null associations, while others have observed non-linear relationships depending on age, sex, exposure window, and the specific biomarker evaluated. Such discrepancies likely reflect differences in study design, exposure assessment, population characteristics, and the complex nature of real-world exposure to multiple endocrine-disrupting chemicals [116,140].

Increasing attention is being paid to prenatal exposure as well. Prospective birth cohort studies have further reported associations between maternal concentrations of bisphenols and phthalates and alterations in the hormonal profile and developmental trajectory of male offspring. These findings further support the hypothesis that prenatal exposure to endocrine disruptors can lead to endocrine dysregulation [50,96,141].

The interpretation of human studies is made difficult by a number of issues. Individuals are exposed to mixtures of chemicals rather than to one chemical, and exposure differs from population to population and even from one time period to another. Furthermore, lifestyle factors, obesity, metabolic disorders, smoking, age, and socioeconomic status may influence both EDC exposure and endogenous androgen concentrations, thereby acting as potential confounders in epidemiological analyses [142,143,144,145]. Another important limitation is that circulating testosterone concentrations represent only one component of androgen function and may not fully reflect tissue-specific androgen activity or receptor signaling. Furthermore, the cross-sectional design of many epidemiological studies precludes determination of temporal relationships between exposure and endocrine alterations. Consequently, current human evidence supports an association between plastic-derived EDC exposure and altered testosterone homeostasis but does not establish causality.

Though there are weaknesses in the findings, the total amount of evidence provided implies that there is an association between long-term exposure to endocrine disruptors formed by plastics and the inability to control testosterone levels [15,66,146].

### 6.3. Effects on Puberty and Adult Androgen Status

Testosterone is the male sex hormone that is primarily responsible for the development of secondary sex characteristics, reproduction, muscle growth, and sexual behavior. Any disruption in the androgen pathway may result in problems with reproductive and metabolic well-being in the future [147,148,149].

Experimental animal studies suggest that EDC exposure during development may alter pubertal timing through disruption of androgen signaling. Human epidemiological studies have likewise reported associations between bisphenol and phthalate exposure and altered pubertal timing; however, findings remain inconsistent across populations. Some studies have reported delayed pubertal development and alterations in reproductive hormone concentrations among exposed individuals, whereas others have observed only modest endocrine changes or no significant associations [107,150,151].

Human epidemiological studies suggest that long-term exposure to plastic-derived EDCs may impair androgen status by interfering with steroidogenic pathways and hormonal homeostasis. The presence of reduced amounts of testosterone has been linked to several conditions, such as low sex drive, poor sexual performance, muscle wasting, fat accumulation, and metabolic problems, among others [3,11,121,152].

The association between EDC exposure and adult testosterone deficiency is likely multifactorial and may involve several of the physiological mechanisms described above. This intricate interaction may contribute to the formation of an environment where the risk of developing hypogonadism increases [13]. Despite increasing evidence, several important uncertainties remain. It is still unclear whether chronic low-dose environmental exposure produces clinically meaningful reductions in testosterone across the general population or whether susceptibility is restricted to vulnerable individuals with genetic, metabolic, or developmental risk factors. Clarifying these issues will require well-designed longitudinal studies with repeated exposure assessment and standardized endocrine outcomes.

It is significant to note that the age-related decline in testosterone levels may be accelerated due to lifetime environmental exposures. Lifetime exposure to plastic-derived contaminants may contribute to the progressive deterioration of endocrine function, particularly among individuals with additional metabolic or genetic risk factors [153].

Collectively, the available literature supports the biological plausibility that plastic-derived EDCs may influence testosterone homeostasis throughout the life course. The evidence is the strongest in experimental models, where consistent alterations in steroidogenesis, Leydig cell function, and endocrine signaling have been demonstrated under controlled conditions [73,154]. In contrast, human epidemiological studies have generally reported associative rather than causal relationships, with considerable heterogeneity arising from differences in exposure assessment, study populations, and potential confounding factors. Consequently, although current findings support concern regarding the endocrine effects of plastic-derived contaminants, additional longitudinal epidemiological studies and mechanistic clinical investigations are needed to clarify the magnitude, timing, and clinical significance of these associations and to bridge the gap between experimental observations and human health outcomes.

A summary of the available evidence linking exposure to plastic-derived endocrine disruptors with testosterone dysregulation across different stages of life is presented in Table 3.

## 7. ED as a Potential Downstream Consequence of Endocrine Disruption

Among the most frequent sexual disturbances experienced by men is ED, which is characterized by the inability to obtain and sustain an adequate erection for successful sexual intercourse. Traditionally linked to conditions such as aging, cardiovascular conditions, diabetes mellitus, and psychological problems, ED is increasingly seen to be caused by other external factors as well. The importance of testosterone for proper erectile functioning and the known disruptive effects of plastic-derived toxins on the endocrine system have made scientists investigate the possible connection between EDCs and ED [11,91,155,156].

Penile erection is a complex neurovascular process requiring coordinated interactions between autonomic nerves, endothelial cells, vascular smooth muscle, and hormonal signaling. Nitric oxide released from both neuronal nitric oxide synthase (nNOS) and endothelial nitric oxide synthase (eNOS) initiates smooth muscle relaxation and penile vasodilation. Testosterone contributes to this process by maintaining nitric oxide synthase expression, preserving cavernosal smooth muscle integrity, and supporting normal neural regulation of erectile function. Consequently, disturbances in androgen signaling may impair both vascular and neurogenic components of erection [21,157,158,159,160,161].

Current evidence indicates that the etiology of ED is multifactorial and involves complex interactions among hormonal, vascular, neurological, metabolic, and psychological factors. The effects of plastic-derived EDCs on erectile function are likely multifactorial, involving not only reductions in testosterone levels but also disruptions of vascular, inflammatory, and metabolic pathways [157,158].

### 7.1. Physiological Role of Testosterone in Erectile Function

Testosterone is required for proper male sexual development, sexual drive, and erectile function. Although penile erection is achieved through vascular and nervous control, sufficient androgen signaling is needed to ensure normal functioning of the penis tissues [20,159]. Testosterone regulates several molecular pathways essential for erectile physiology, including nitric oxide synthase expression, endothelial function, smooth muscle integrity, and phosphodiesterase type 5 signaling, thereby facilitating normal penile vasodilation and erectile function [21,159].

Several experimental studies have shown that androgen deficiency induces structural alterations in the corpus cavernosum, including smooth muscle loss, increased fibrosis, endothelial dysfunction, and impaired vasodilation. These structural and functional changes impair penile hemodynamics, thereby contributing to erectile dysfunction. In men with hypogonadism, testosterone replacement therapy has been shown to improve erectile function, libido, and other aspects of sexual health, particularly in those with documented androgen deficiency [21,159,160].

Most evidence supporting this mechanism originates from experimental studies demonstrating disruption of androgen signaling and penile tissue structure. Although testosterone deficiency is a recognized contributor to erectile dysfunction, direct evidence that environmentally relevant EDC exposure produces clinically significant androgen deficiency leading to ED in humans remains limited [15,161].

### 7.2. Endothelial Dysfunction and Nitric Oxide Signaling

Erectile function depends on the relaxation of corpus cavernosum smooth muscle through endothelium-dependent mechanisms, resulting in vasodilation and increased penile blood flow. A key process involved in this response is NO-mediated signaling and cyclic guanosine monophosphate signal transduction, which regulate smooth muscle relaxation and penile erection [162,163].

Emerging evidence suggests that exposure to plastic-derived EDCs may adversely affect endothelial function. Experimental studies have demonstrated that BPA and phthalates can impair eNOS activity, reduce NO bioavailability, and promote OS, thereby contributing to endothelial dysfunction [164,165,166]. However, these findings are derived predominantly from experimental models. Whether comparable endothelial alterations occur following chronic low-dose environmental exposure in humans remains incompletely understood.

Endothelial dysfunction is widely recognized as one of the earliest manifestations of vascular disease. Since the penile vasculature is highly dependent on intact endothelial function and NO signaling, even mild endothelial impairment may compromise penile blood flow and contribute to erectile dysfunction [167].

### 7.3. OS, Inflammation, and Vascular Damage

The involvement of OS as a major pathological process behind endocrine disruption, as well as ED, has been proposed recently. Excessive ROS production can damage endothelial cells, impair NO signaling, induce vascular inflammation, and promote tissue remodeling within erectile tissues [23,168].

Experimental and observational studies suggest that exposure to plastic-derived contaminants may contribute to OS in vascular tissues relevant to erectile function. Elevated ROS levels may decrease the availability of NO due to their interactions while simultaneously increasing the activity of inflammatory cascade processes [169].

Inflammation may represent an additional mechanism contributing to vascular injury, leading to endothelial activation, smooth muscle dysfunction, and structural alterations of the penile arteries. As a result, vascular sensitivity will decline, and the ability to sustain erections becomes compromised [170,171,172].

Finally, OS may further compromise vascular and smooth muscle function within erectile tissues. Considering that erectile physiology involves vascular and smooth muscle functions, oxidative damage to mitochondria may add another problem in erectile disorders after EDC exposure [23]. Although OS and inflammation are consistently implicated in experimental studies of EDC toxicity, both processes represent common pathological pathways shared by numerous chronic diseases. Therefore, their presence alone does not establish a causal relationship between plastic-derived EDC exposure and erectile dysfunction in human populations.

### 7.4. Clinical and Epidemiological Evidence Linking EDCs to ED

Despite there being a rich body of research exploring reproductive toxicity and testosterone disruption, the connection between EDC exposure and erectile dysfunction has not yet been extensively studied. However, a significant number of findings suggest the biological plausibility of the suggested link [11,117].

Human epidemiological studies evaluating erectile dysfunction as a clinical outcome following exposure to plastic-derived EDCs remain relatively limited. Most observational studies have examined reproductive hormone concentrations or markers of male reproductive health rather than clinically diagnosed erectile dysfunction. Although several studies have reported associations between higher BPA or phthalate exposure and adverse reproductive health indicators, direct epidemiological evidence linking EDC exposure to erectile dysfunction remains scarce and largely associative [91,107].

Moreover, most of the biological pathways involved in EDC toxicity are similar to those contributing to ED. Such processes as endothelial dysfunction, OS, chronic inflammation, metabolic disturbances, obesity, insulin resistance, and cardiovascular diseases have been shown to be common in individuals exposed to environmental contaminants as well as experiencing ED [173,174,175].

Metabolic dysfunction may represent an important indirect pathway linking EDC exposure with erectile dysfunction. Many plastic-derived EDCs have been classified as obesogens because they promote adipose tissue accumulation, insulin resistance, dyslipidemia, and metabolic syndrome. Since these conditions are well-established risk factors for endothelial dysfunction and erectile dysfunction, they may partially mediate the observed associations between environmental EDC exposure and impaired sexual function [176,177,178].

Despite the limited evidence currently available in the literature to support a direct association, the existence of such an effect is biologically plausible based on the endocrine, vascular, inflammatory, and metabolic pathways involved. Further research is warranted to clarify this potential relationship [179,180].

Overall, ED may represent a downstream consequence of lifelong exposure to plastic-derived endocrine-disrupting chemicals through multiple interconnected hormonal, vascular, neurovascular, inflammatory, and metabolic mechanisms [3]. However, while experimental studies provide strong biological plausibility, direct human epidemiological evidence remains limited and largely associative [154]. Consequently, further longitudinal epidemiological studies and mechanistic clinical investigations are needed to clarify whether chronic environmental exposure to plastic-derived EDCs contributes directly to erectile dysfunction in humans.

**Figure 2:**
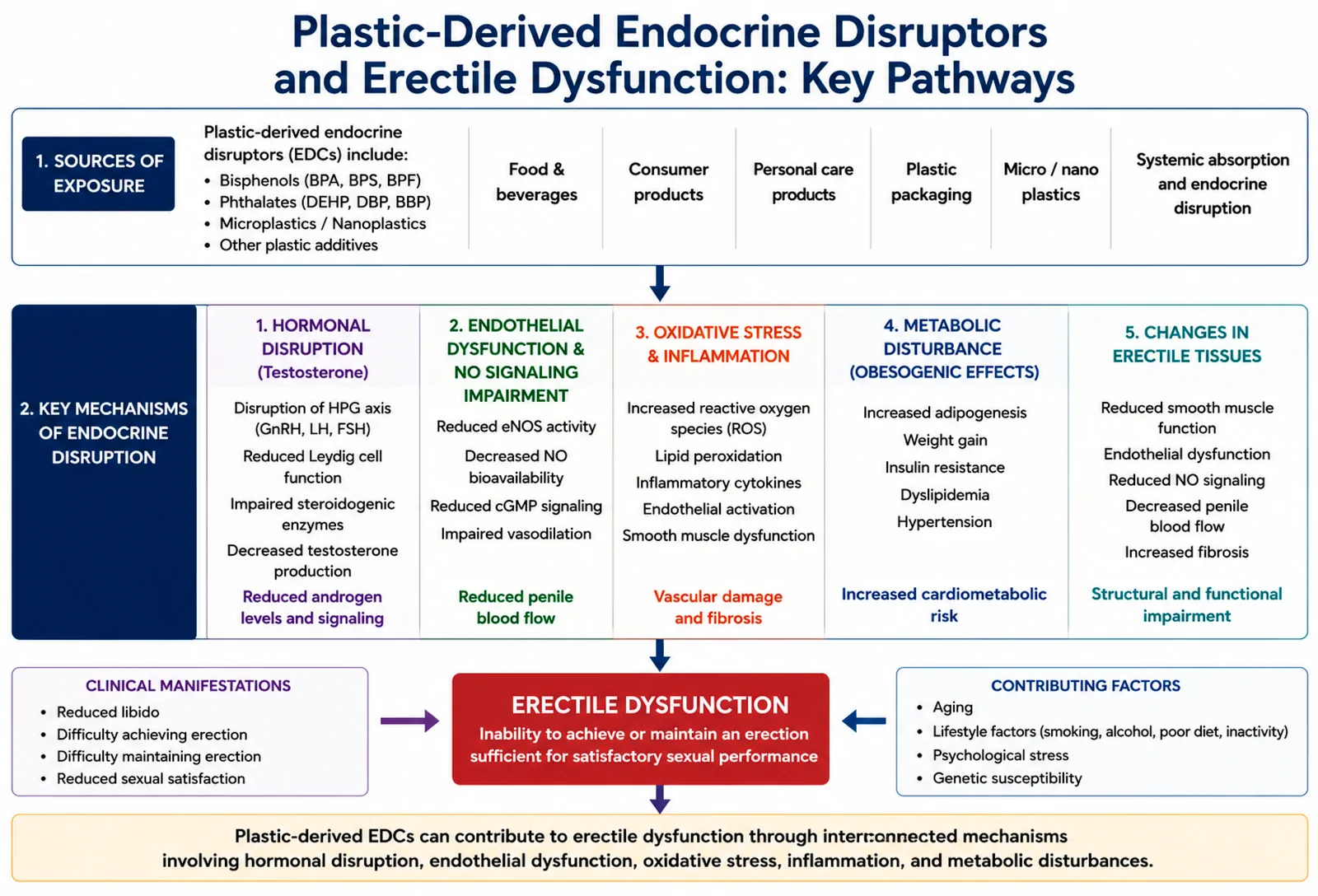
Proposed pathways through which plastic-derived endocrine disruptors may contribute to erectile dysfunction. Exposure to plastic-derived EDCs may promote erectile dysfunction through endocrine disruption, endothelial dysfunction, oxidative stress, inflammation, metabolic dysregulation, and structural changes in erectile tissues. These mechanisms may reduce androgen signaling, impair nitric oxide-mediated vasodilation, decrease penile blood flow, and compromise erectile function, ultimately contributing to the development of erectile dysfunction.

The proposed pathways linking prenatal exposure to plastic-derived endocrine disruptors with adverse male reproductive outcomes throughout the life course are summarized in Figure 2.

## 8. Emerging Concerns: Microplastics, Nanoplastics, and Male Sexual Health

The increasing accumulation of plastic waste worldwide has raised concerns about the potential health consequences of exposure to microplastics and nanoplastics [154]. Early research on endocrine disruptors primarily focused on the health effects of plastic-associated chemicals such as bisphenols and phthalates. More recently, attention has shifted toward the plastic particles themselves [15]. Microplastics and nanoplastics are increasingly recognized as emerging environmental contaminants with the potential to affect biological systems through both the release of associated chemicals and direct particle–cell interactions. However, compared with bisphenols and phthalates, the evidence regarding their endocrine and reproductive effects remains considerably less mature, with most available data derived from experimental models [181,182].

Their small size allows microplastics and nanoplastics to be readily ingested or inhaled, enabling systemic distribution and accumulation in various tissues. The discovery of microplastics and nanoplastics in a variety of human organs, including reproductive cells, emphasizes how frequently and continuously people are exposed to these substances [41,183].

In contrast to many EDCs, microplastics may influence biological processes through both particle-mediated and chemical-mediated mechanisms [184]. Numerous environmental pollutants, such as bisphenols, phthalates, heavy metals, polycyclic aromatic hydrocarbons (PAHs), and persistent organic pollutants (POPs), can be transported via microplastics. Through this mechanism, microplastics may enhance the toxicity of endocrine-active substances by increasing their bioavailability. As a result, human exposure frequently involves multiple environmental contaminants occurring simultaneously [26,185].

A growing body of experimental evidence has begun to elucidate the mechanisms through which microplastics and nanoplastics may impair male reproductive function [53,135,186]. Experimental studies have reported particle accumulation within reproductive tissues, disruption of the blood–testis barrier, impaired steroidogenesis, Sertoli and Leydig cell dysfunction, oxidative stress, mitochondrial dysfunction, inflammation, apoptosis, altered cell signaling, and impaired spermatogenesis. Collectively, these mechanisms may contribute to reduced testosterone biosynthesis and compromised male reproductive function [135,187].

Nanoplastics are particularly worrisome because of the possibility that they will get inside cells and interact with intracellular structures. In particular, some researchers have reported the ability of nanoplastics to localize inside mitochondria, causing disturbance of cell homeostasis, ROS production, and changes in gene expression. It is likely that these changes will reduce the production of testosterone by interfering with the functioning of Leydig cells [188]. Because of their nanoscale dimensions, nanoplastics may also enter intracellular organelles more readily than larger microplastics, potentially resulting in greater biological reactivity.

Evidence is accumulating on the possibility that both microplastics and nanoplastics could cause developmental toxicity. By disrupting the development process dependent on androgens, the developmental process of reproduction could be affected when such nanoparticles and microplastics are exposed to embryos during pregnancy. Androgens are vital for embryonic reproductive development [189,190].

Although the production of reproductive hormones is one of the possible health effects of microplastics, several physiological mechanisms connected to the pathogenesis of ED might be influenced by biological changes caused by microplastics as well [191,192]. Current evidence does not directly link microplastic exposure with ED, and epidemiological studies evaluating this association remain scarce. Instead, the proposed relationship is inferred from experimental evidence demonstrating endocrine disruption, endothelial dysfunction, OS, and inflammatory pathways known to contribute to erectile physiology. Consequently, the association should currently be regarded as biologically plausible rather than clinically established [26,53,191].

Although increasing attention has been devoted to this topic, research on the effects of microplastics on the human reproductive and endocrine systems remains limited, and standardized methods for assessing individual exposure have yet to be established. Furthermore, the heterogeneity of the available findings may be attributed to differences in particle size, polymer composition, shape, surface characteristics, concentration, exposure route, and duration of exposure [135,186,191]. Additionally, comparison among studies is complicated by substantial methodological heterogeneity, including differences in particle size, polymer composition, shape, surface chemistry, concentration, exposure route, duration of exposure, and analytical methods used for particle detection. These limitations currently hinder direct comparison between studies and complicate translation of experimental findings to human health risk assessment.

Overall, current evidence suggests that microplastics and nanoplastics may impair male reproductive health through both particle-mediated and chemical-mediated mechanisms involving endocrine disruption, OS, inflammation, mitochondrial dysfunction, and impaired steroidogenesis. However, most evidence derives from experimental models, whereas human data remain limited. Further epidemiological and mechanistic studies are needed to establish their clinical significance [26,51].

## 9. Prevention Strategies and Regulatory Perspectives

It is alarming for public health that there are so many EDCs and microplastics from plastics present in the environment. Taking into consideration how EDCs and microplastics can affect testosterone levels and sexual health, preventive actions must be undertaken by the individual and society. Although it may not be possible to fully protect oneself from EDCs and microplastics, increased knowledge is needed [2,3,79,183].

### 9.1. Individual-Level Exposure Reduction Strategies

There are several practices that would minimize exposure to plastic-related chemicals. Dietary intake represents one of the primary routes of human exposure to bisphenols, phthalates, and microplastics. Accordingly, reducing the use of plastic food packaging, avoiding the heating of food in plastic containers, limiting the consumption of highly processed foods, and choosing fresh products may help reduce exposure to these contaminants [33,45,183].

Individuals may also reduce exposure by using glass, stainless steel, or ceramic containers instead of plastic containers for food and beverages. Additional strategies to reduce exposure include minimizing contact with thermal paper receipts, selecting phthalate-free products, and maintaining good indoor air quality. In addition, as household dust can serve as a reservoir of plastic-related contaminants, regular cleaning and adequate ventilation may help reduce exposure [2,79].

Special emphasis must be placed on vulnerable groups such as pregnant women, newborn babies, children, and teenagers. As early-life exposure may have long-term implications for reproductive health, preventive action during pregnancy may be particularly important. While definite clinical guidelines are yet to emerge, an approach that seeks to avoid unnecessary exposure seems reasonable [93,96,101].

### 9.2. Public Health and Regulatory Actions

The potential health effects of EDCs have become a major focus of regulatory authorities worldwide. Over the past two decades, numerous countries and international organizations have implemented regulatory measures to restrict the use of BPA, certain phthalates, and other endocrine-disrupting chemicals in food-contact materials, infant and childcare products, medical devices, and consumer goods [3,54,79].

There are many things that are yet to be accomplished in this regard. One of the major difficulties lies in the use of structurally similar compounds as substitutes for prohibited compounds, which have not been evaluated for their potential impacts. The case of BPS and BPF is an example of such regrettable substitution [4,28,31].

Current regulatory frameworks face challenges in assessing the cumulative effects of exposure to chemical mixtures. Human exposure generally involves mixtures of EDCs rather than individual compounds, with the potential for additive or synergistic biological effects. Consequently, traditional risk assessment approaches that evaluate chemicals individually may underestimate the risks associated with combined exposures [120,142,144].

The growing problem of microplastics and nanoplastics adds to the list of regulatory challenges. Methods of assessing exposure, conducting toxicology studies, and monitoring environments are still in development. Standardization of criteria for detecting plastic particles will be crucial in the future for conducting risk assessments [7,38,181].

### 9.3. Clinical Awareness and Risk Communication

Healthcare providers should be encouraged to raise awareness of environmental exposures that may influence reproductive and sexual health outcomes. Traditionally, the study of testosterone insufficiency, infertility, and erectile dysfunction was concentrated on the role of genetic, hormonal, metabolic, and lifestyle factors. There is growing recognition that environmental exposures should be considered alongside traditional risk factors affecting reproductive and sexual health [11,54,61].

The integration of environmental exposure considerations into reproductive health counseling may aid in identifying potential risks and promoting preventive strategies. Physicians treating men who have hypogonadism, infertility, or erectile dysfunction should keep abreast of emerging research that shows how the endocrine-disrupting chemicals affect reproductive health. The same can be done during preconception and prenatal counseling [61,78,93].

The importance of effective risk communication should not be overlooked. It is necessary to provide people with correct information about the state of scientific research on the subject without causing any undue panic. Given the existing uncertainties surrounding safe exposure levels and long-term health consequences, the precautionary principle advocates limiting unnecessary exposure to endocrine-disrupting chemicals [79,95].

Addressing the reproductive health consequences of exposure to plastic-associated chemicals will require a multidisciplinary and coordinated approach involving all relevant stakeholders. Enhanced chemical safety evaluation, comprehensive environmental monitoring, and evidence-based interventions are critical for safeguarding the health of future generations [2,54,79].

## 10. Current Limitations and Future Directions

### 10.1. Limitations of Current Evidence

Despite the growing body of evidence linking plastic-derived endocrine-disrupting chemicals to alterations in testosterone homeostasis and male reproductive health, several important limitations should be acknowledged. Much of the available evidence is derived from in vitro experiments and animal studies, whereas human data are predominantly observational and frequently based on cross-sectional designs, limiting causal inference. In addition, substantial heterogeneity exists in exposure assessment methods, biomonitoring approaches, study populations, and outcome measures, making direct comparisons across studies challenging. These limitations underscore the need for standardized methodologies, longitudinal investigations, and mechanistic clinical studies to better define the relationship between plastic-derived endocrine disruptors, testosterone dysregulation, and ED [61,78,154].

### 10.2. Future Research Priorities

Nonetheless, despite the substantial increase in studies investigating EDCs, considerable uncertainty remains regarding their long-term effects on testosterone homeostasis and male reproductive health. Although experimental and laboratory studies have demonstrated that plastic-derived chemicals may influence endocrine system function, their clinical significance has not yet been fully established. Therefore, further research is needed to improve exposure assessment methodologies, establish causal relationships, and elucidate the biological mechanisms linking exposure to environmental contaminants with testosterone deficiency and erectile dysfunction [61,78,154].

One of the most important priorities for future research is the implementation of large-scale longitudinal studies that follow individuals from birth through adulthood. A major limitation of current epidemiological evidence is the reliance on cross-sectional study designs and single-time-point exposure measurements. Longitudinal investigations incorporating biomonitoring, together with comprehensive hormonal, reproductive, and sexual health assessments, are warranted to provide a more robust understanding of these associations [61,98,143].

In addition, improved exposure assessment methodologies are needed. Although environmental exposure is usually chronic, urinary and serum biomarkers often reflect only short-term exposure. Future studies should incorporate environmentally relevant exposure levels that better reflect real-world human exposure patterns and investigate the potential biological effects of low-dose exposures, including non-monotonic dose–response relationships that may not be predicted by conventional toxicological models. Repeated exposure measurements, cumulative exposure indices, and advanced analytical approaches for assessing chemical mixtures should also be incorporated into future research. The potential additive and synergistic effects of combined exposure to bisphenols, phthalates, microplastics, nanoplastics, and other environmental pollutants require further investigation [142,144,145].

Further research is needed to clarify the effects of microplastics and nanoplastics on reproductive health. Despite the growing body of evidence from animal studies, human data on the reproductive toxicity of these particles remain limited. The development of standardized methodologies for the detection, characterization, and quantification of plastic particles is essential to improve comparability across studies. In addition, additional research is needed to determine whether microplastics directly disrupt endocrine function or act primarily as carriers of other environmental pollutants [7,38,181].

The biochemical basis for EDC-induced dysregulation of testosterone needs further examination. The biochemical processes underlying endocrine dysregulation need more consideration. The development in proteomic and genomics technologies, especially high-throughput sequencing, may aid in discovering new biomarkers that can help identify molecular changes before the appearance of any clinical symptoms [22,83,154].

More research is needed to elucidate the link between endocrine disruption and erectile dysfunction. So far, there has been little research done on this link. Future studies need to conduct a comprehensive analysis of hormones, erectile dysfunction, as well as vasculature and metabolism [155,192].

Further studies need to be carried out among sensitive populations. Individual sensitivity to the impacts of endocrine disruptors may be dependent upon various factors, including genetics, lifestyle factors, nutrition, obesity, and co-morbid conditions. Such knowledge may be useful for the design of individual preventive measures [93,96,107].

To determine the effectiveness of treatment methods aimed at reducing the exposure levels of these toxins, more research must be done. Proof that there have been positive changes in the endocrine and reproductive functions due to decreased exposure will provide evidence of causation. The future of this area of study will also require collaboration between experts from different disciplines [2,79,154].

In conclusion, a holistic view of the impacts of plastic pollutant exposure on the regulation of testosterone involves bringing together the information from various studies. In addition to improving our understanding of the impacts of environmental pollution on reproduction, such research will help to create measures that protect reproductive health among men [54,61,154].

## 11. Conclusions

Male reproductive and sexual health may be adversely affected by exposure to plastic-derived contaminants, including phthalates, bisphenols, and contaminants associated with micro- and nanoplastics. Current evidence supports the biological plausibility that these endocrine-disrupting chemicals may interfere with testosterone homeostasis and male reproductive function through multiple interconnected mechanisms.

However, much of the available human evidence is associative and originates from observational epidemiological studies, whereas most mechanistic insights derive from in vitro experiments and experimental animal models. Consequently, the causal pathways linking chronic environmental exposure to plastic-derived endocrine-disrupting chemicals with testosterone dysregulation, erectile dysfunction, and impaired reproductive health remain incompletely established in humans.

Future research should prioritize well-designed longitudinal epidemiological studies, improved exposure assessment methods, the identification of reliable biomarkers, and mechanistic clinical investigations to clarify the magnitude, timing, and clinical significance of these associations. Such evidence will be essential for developing evidence-based preventive strategies to preserve male reproductive health.

## Figures and Tables

**Table 1:** Major mechanisms through which plastic-derived endocrine-disrupting chemicals affect male reproductive function.

Mechanism	Main Targets	Representative EDCs	Potential Consequences
Disruption of the HPG axis [12,14,62]	Hypothalamus, pituitary gland, Leydig and Sertoli cells	BPA, phthalates	Altered GnRH, LH, FSH, and testosterone secretion; impaired reproductive development
Impaired steroidogenesis and Leydig cell dysfunction [18,19,67]	StAR, CYP11A1, 3β-HSD, 17β-HSD, Leydig cells	BPA, phthalates	Reduced testosterone synthesis and androgen deficiency
AR interference [30,72,73]	AR signaling pathways	Phthalates, BPA and related bisphenols	Impaired masculinization, altered spermatogenesis, sexual dysfunction
OS and mitochondrial dysfunction [22,26,69]	Mitochondria, antioxidant defense systems	BPA, phthalates, microplastics, nanoplastics	ROS overproduction, cellular damage, apoptosis, impaired testicular function
Epigenetic modifications [82,83,90]	DNA methylation, histone modifications, non-coding RNAs	BPA, phthalates, other plastic-associated EDCs	Altered gene expression, long-term reproductive effects, potential transgenerational inheritance

Abbreviations: AR, androgen receptor; BPA, bisphenol A; CYP11A1, cholesterol side-chain cleavage enzyme (cytochrome P450 family 11 subfamily A member 1); DNA, deoxyribonucleic acid; EDCs, endocrine-disrupting chemicals; FSH, follicle-stimulating hormone; GnRH, gonadotropin-releasing hormone; HPG, hypothalamic–pituitary–gonadal; HSD, hydroxysteroid dehydrogenase; LH, luteinizing hormone; OS, oxidative stress; RNAs, ribonucleic acids; ROS, reactive oxygen species; StAR, steroidogenic acute regulatory protein.

**Table 2:** Effects of prenatal exposure to plastic-derived endocrine disruptors on male reproductive development and long-term reproductive health.

Developmental Stage	Main Targets	Potential Effects of EDC Exposure	Possible Long-Term Consequences
Placental transfer [99,101,102]	Placenta, placental hormones, nutrient transport systems	Placental passage of EDCs, altered hormone production, oxidative stress, inflammation	Modified intrauterine environment
Fetal testicular development [18,103,110]	Leydig cells, Sertoli cells, steroidogenic pathways	Reduced testosterone production, impaired Leydig cell differentiation, altered steroidogenic gene expression	Disrupted masculinization
Masculinization programming window [104,106,115]	Androgen-dependent developmental processes	Reduced androgen action during critical developmental periods	Cryptorchidism, hypospadias, decreased AGD
Developmental programming of androgen production [14,109,111]	HPG axis, Leydig cells, epigenetic regulators	Persistent alterations in steroidogenesis and endocrine regulation	Testosterone deficiency and endocrine dysfunction
Postnatal and adult life [61,107,118]	Reproductive and endocrine systems	Impaired spermatogenesis, hormonal imbalance, reduced semen quality	Infertility, sexual dysfunction, reproductive disorders
Transgenerational effects [88,90,123]	Epigenetic mechanisms	Heritable epigenetic alterations	Potential reproductive abnormalities in subsequent generations

Abbreviations: AGD, anogenital distance; EDC, endocrine-disrupting chemical; HPG, hypothalamic–pituitary–gonadal.

**Table 3:** Evidence linking plastic-derived endocrine disruptors to testosterone dysregulation across the lifespan.

Exposure Category	Main Findings	Proposed Mechanisms	Reported Outcomes
Experimental animal studies [18,67,146]	Reduced testosterone levels following BPA, BPS, BPF, and phthalate exposure	Impaired steroidogenesis, Leydig cell dysfunction, OS, altered gene expression	Testosterone deficiency, reproductive dysfunction
Human epidemiological studies [116,131,145]	Associations between BPA/phthalate biomarkers and altered hormone profiles	Endocrine disruption, hormonal dysregulation, mixture effects	Reduced testosterone levels, altered reproductive hormones
Prenatal exposure [46,93,96]	Hormonal alterations in offspring and developmental disturbances	Developmental programming, epigenetic modifications, HPG-axis disruption	Reduced AGD, altered reproductive development, endocrine dysfunction
Pubertal exposure [85,150,151]	Changes in pubertal timing and hormone concentrations	Interference with androgen signaling	Delayed or altered pubertal development
Adult and lifelong exposure [61,107,153]	Chronic disruption of testosterone homeostasis	Steroidogenic impairment, OS, cumulative endocrine effects	Hypogonadism, sexual dysfunction, metabolic disturbances

Abbreviations: AGD, anogenital distance; BPA, bisphenol A; BPF, bisphenol F; BPS, bisphenol S; HPG, hypothalamic–pituitary–gonadal; OS, oxidative stress.

## Data Availability

No new data were created or analyzed in this study. Data sharing is not applicable to this article.
